# Diversity and functional characterization of HNH endonucleases encoded by lactococcal Skunavirus phages

**DOI:** 10.1099/mgen.0.001548

**Published:** 2025-12-23

**Authors:** Jun-Hyeok Yu, Christian Cambillau, Gabriele Andrea Lugli, Marco Ventura, Arjen Nauta, Jennifer Mahony, Douwe van Sinderen

**Affiliations:** 1 School of Microbiology & APC Microbiome Ireland, University College Cork, Cork, Ireland; 2 Laboratoire d’Ingénierie des Systèmes Macromoléculaires (LISM), Institut de Microbiologie, Bioénergies et Biotechnologie (IMM), Aix-Marseille Université – CNRS, UMR 7255, Marseille, France; 3 Department of Chemistry, Laboratory of Probiogenomics, Life Sciences and Environmental Sustainability, University of Parma, Parma, Italy; 4 Interdepartmental Research Centre "Microbiome Research Hub", University of Parma, 43124 Parma, Italy; 5 FrieslandCampina, Amersfoort, The Netherlands

**Keywords:** bacteriophage, comparative genomic analysis, evolution, homing endonuclease, *Lactococcus*, HNH endonuclease

## Abstract

Homing, a biological phenomenon involving enzyme-mediated genetic exchange by homologous recombination, has been highlighted as a potential driver of phage genome evolution. In the current study, 18 lactococcal phages, belonging to the *Skunavirus* genus, were isolated from Dutch dairy facilities, and their genomes were sequenced. Together with 71 phages from previous studies involving Dutch dairy fermentation facilities, a total of 89 *Skunavirus* genomes were analysed, revealing a strong correlation between phage diversity and the applied starter culture. These analysed *Skunavirus* genomes were predicted to encode a total of 212 intact HNH endonucleases (HNHEs), which were classified into families based on structural homology and their insertion locations on the genome. Members of the I-HmuI-like HNHE family were observed to be present among most analysed genomes, though they varied in individual *Skunavirus* phages in both their number and genomic locations. Phylogenetic analysis revealed that these I-HmuI-like HNHEs cluster together according to their insertion locations and the corresponding starter cultures. Furthermore, the so-called genetic marker exclusion activity of particular expressed HNHEs against *Skunavirus* sk1 infection was observed, indicative of their role in phage genome evolution and associated adaptation processes.

**Impact Statement**

Bacteriophages (or phages), viruses that infect bacteria, are regarded as a major risk factor in the dairy industry, as they may delay or completely disrupt milk fermentations. Although various strategies have been implemented to reduce the impact of phage infections, the genomic diversity and virulence of these phages continue to challenge the complete prevention of their propagation during fermentation. However, our understanding of the underlying factors driving this variability remains limited. In this study, we examined the genomic diversity of phages isolated from dairy samples collected from various environments and under different conditions (i.e. location of isolates, used starter culture and year of isolation). Furthermore, we explored the role of homing, a process that facilitates genetic exchange between phages, in contributing to this diversity. Focusing on phage-encoded HNH endonucleases as potential homing vehicles, we characterized their prevalence, genomic locations and protein sequence similarities. Our findings support the notion that HNH endonucleases instigate diversification and evolutionary adaptation of bacteriophages in dairy environments.

## Data Summary

The genome sequences of the phages used in this study are available in the National Center for Biotechnology Information (NCBI) database under accession numbers listed in Table S3.

## Introduction

Bacteriophages (or phages) play a crucial role in the biosphere, shaping the functional and taxonomic composition and the dynamics of microbial communities [1,2]. In response to the selective pressure of phage predation, bacteria have evolved numerous defence strategies, which act at different stages of the phage life cycle [3,5]. However, phages in turn adapt and evolve to overcome these bacterial anti-phage systems, thereby facilitating co-evolution of both bacterial and associated phage communities [6,8]. The genetic adaptations of phages infecting bacteria that make up starter cultures used in dairy fermentation may not only result in increased lytic virulence with a broader host range, shorter latency time and/or higher burst size, but also in enhanced resistance against thermal/biocidal treatments, posing significant challenges for the dairy fermentation industry [9,13]. Owing to the economic importance of fermented foods and their associated starter cultures, various studies related to phage diversity in fermentation facilities and associated foods have been conducted to identify factors that underpin phage genetic variation [14,17].

**Fig. 1.**
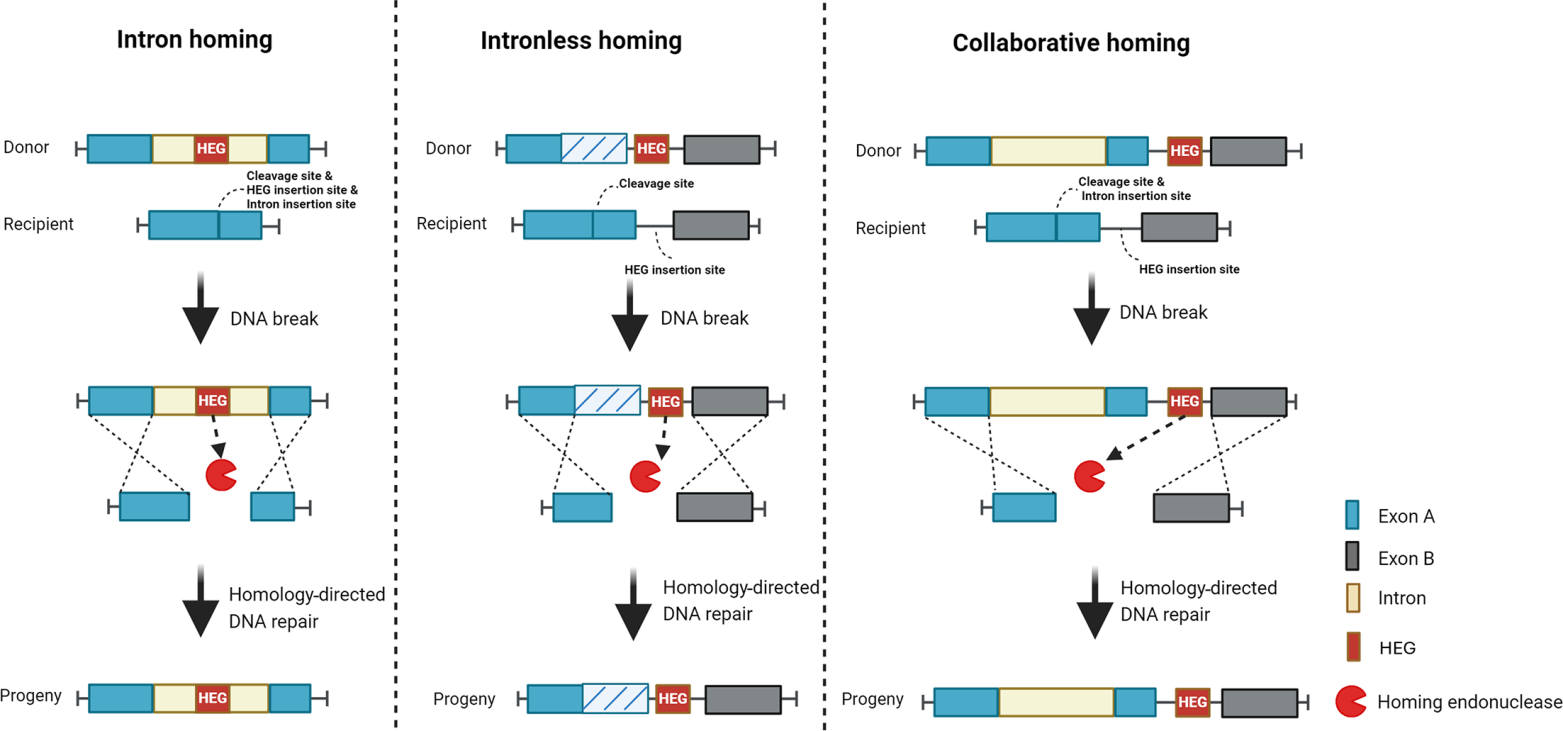
Schematic overview of homing mechanisms mediated by endonucleases. *Exon with pattern fills indicates DNA sequence polymorphism.

Recently, the genetic phenomenon designated as ‘homing’ has been proposed as a driver of genetic exchange between phages [18]. Homing, first observed in *Saccharomyces cerevisiae* and involving unidirectional inheritance of the mitochondrial ω allele, was originally described as a phenomenon in which a so-called homing endonuclease-encoding gene (HEG) is transferred along with an insertion sequence (self-splicing intron or intein) to a cognate allele lacking the sequence [19,20]. However, instances of homing, in which the transfer of the HEG is not associated with an intron sequence, have also been described (Fig. 1) [21]. Consequently, homing is classified as follows: (i) intron homing, in which the HEG is located within an intron and transferred along with the intron; (ii) intronless homing, in which the HEG exists as a ‘free-standing’ gene (i.e. not subject to mRNA splicing) and (iii) collaborative homing, in which the HEG is a free-standing gene yet transferred along with a separate intron [22].

Homing is facilitated by so-called homing endonucleases, which are encoded by the donor DNA molecules and which generate a sequence-specific, double-stranded DNA break in the homologous allele of the recipient DNA molecule, typically within an essential host gene [23]. Transfer of the HEG from the donor to the recipient is then accomplished through repair of the cleaved recipient DNA strand through homologous recombination with the donor strand [24]. In the case of homing where the HEG is not encoded within an intron, a free-standing HEG is inserted into a location unconnected to the gene containing the target cleavage sequence [21,25]. Also, the recipient DNA molecule, which acquires the HEG, is assumed to become resistant to the corresponding homing endonuclease because the cleavage sequence of the recipient is replaced by an alternative sequence originating from the donor allele (Fig. 1) [21].

HEGs are frequently present in phage genomes, and several studies have explored their roles [18,26, 27]. Notably, the free-standing HEGs in phage T4 have been studied extensively [21,28,30]. In mixed infections of T4 and T2, the homing endonuclease encoded by T4 can selectively exclude genetic markers of T2 that lack the corresponding HEG, in a process referred to as ‘marker exclusion’, facilitating the generation of a T2-derived progeny that contain certain T4 genetic markers [30]. Furthermore, frequent transfer of genes adjacent to the homing endonuclease cleavage site was observed, likely due to the physical distance between the HEG insertion site and the cleavage site. This suggests that while homing is involved in HEG transfer, it may also facilitate the transfer of flanking genetic material, thus serving as a driver of genetic exchange.

Members of the HNH endonuclease (HNHE) family, deriving their name from the H-N-H/N amino acid (AA) motif that constitutes their active site, are known to function as restriction enzymes, bacterial toxins or phage DNA-packaging aides [31,33]. The homing HNHE is recognized to constitute the largest group of the HNHE family [24]. The structural and enzymatic features have been extensively investigated among various HNHEs (i.e. ColE7, I-HmuI, PacI, Hpy99I, *Geobacter metallireducens* GS-15 HNHE and GVE2) [31,32, 34,37]. In the genome of lactococcal phages, particularly among members of the *Skunavirus* genus, genes predicted to encode homing endonucleases of the HNHE family are commonly identified [26,38, 39]. Members of the *Skunavirus* genus in lactococcal phages are particularly problematic in the dairy industry due to their adaptability and virulence [11,40]. Despite the common presence of HNHE-encoding genes in the genomes of the *Skunavirus* members, their precise activity and genetic consequences remain unexplored [26,38, 39].

In the current work, the presence, diversity and activity of homing endonucleases in lactococcal *Skunavirus* phages were investigated. A total of 89 *Skunavirus* genomes isolated from several Dutch dairy facilities, including 18 newly isolated phages, were analysed. A number of HNHEs encoded in *Skunavirus* genomes were discovered, and their insertion locations within the genome and structural characteristics were assessed. As previously described, genetic variability of *Skunavirus* genomes was highly associated with their bacterial host rather than the location of isolation or sampling time. Also, the phylogenetic clustering of these HNHEs was specific to their genomic insertion locations as well as the corresponding starter cultures used for fermentation. Additionally, genetic marker exclusion activity of HNHEs against *Skunavirus* sk1 infection was examined *in vivo*, in some cases supporting their presumed role in driving genetic exchange between phages via homing during coinfection.

## Methods

### Sample pretreatment and bacteriophage screening

A total of 103 samples from several dairy fermentation facilities in the Netherlands (Table S1, available in the online Supplementary Material) were subjected to pretreatment prior to phage isolation as described previously [41], and briefly outlined as follows. After thawing frozen samples at room temperature, samples were diluted with cold trisodium citrate (2% w/v) and homogenized using a stomacher (Lab-Blender 400; Gemini, the Netherlands) for 5 min. Large solid particles were removed by centrifugation at 300***g*** for 10 min at 4 °C, followed by a second centrifugation of the supernatant at 4,500***g*** for 50 min at 4 °C. The resulting solution was sequentially filtered through 0.45 and 0.2 µM pore-sized syringe filters. The filtered liquid was diluted five-fold with cold salt magnesuim (SM) buffer (50 mM Tris-HCl [hydrochloride], pH 7.5, 200 mM NaCl [sodium chloride], 10 mM MgSO_4_ [magnesium sulfate]). From the processed samples, bacteriophages were isolated using *Lactococcus cremoris* or *Lactococcus lactis* strains from undefined starter cultures SM1 (strains A–T), SM2 (strains 1–20) [42] and SM3 (strains L1, L2, L6, etc.) [43], as well as lactococcal strains NZ9000 and 3107 as indicators [44,45]. Bacterial cultures were prepared in M17 (Millipore, USA) medium supplemented with 0.5% lactose (LM17) at 30 °C. The presence of phages in a given sample was screened using the double-agar (LM17) method supplemented with CaCl_2_ (calcium chloride; final concentration 10 mM), as described previously [46]. After overnight incubation at 30 °C, if plaques were observed, some of these were used for propagation purposes with the corresponding bacterial host (1% inoculation from an overnight culture) for 3 h at 30 °C in LM17 broth supplemented with 10 mM CaCl_2_.

### Phage DNA preparation and genome sequencing

Phage genomic DNA was extracted from lysates of individually propagated phage (10 ml lysate with a phage titre ranging between 10^8^ and 10^10^ PFU [plaque-forming unit] ml^−1^). Phage particles were first precipitated overnight at 4 °C following the addition of 10% polyethylene glycol (final concentration; PEG8000; Sigma Aldrich, USA). Following phage particle collection by centrifugation at 15,000***g*** for 15 min at 4 °C, the pellet was resuspended in 1 ml of SM buffer. The recovered virion concentrate was treated with DNase (final concentration: 10 U ml^−1^) for 30 min at room temperature, and the reaction was halted by adding 0.5 M EDTA. After proteinase K treatment (final concentration: 250 µg ml^−1^) for 15 min at 55 °C, phage DNA was extracted using the Phage DNA Isolation Kit (Norgen Biotek, Canada) according to the manufacturer’s instructions.

The extracted phage DNA was subjected to phage genotyping using multiplex PCR with primer pairs specific for each of the two major lactococcal phage genera, *Skunavirus* and *Ceduovirus*, as well as for representatives of the P335 phage group (Table S2), as described previously [47]. Subsequently, whole-genome sequencing was performed by GenProbio srl (Parma, Italy) using a MiSeq platform (Illumina, San Diego, CA, USA). The libraries of phage DNA were prepared using an Illumina Nextera XT DNA Library Preparation Kit (Illumina, San Diego, CA, USA), and the paired-end reads (250 bp) were assembled using the SPAdes program v3.14. ORFs were predicted using Prodigal [44], and ORFs were annotated against the National Centre for Biotechnology Information (NCBI) RefSeq database and INTERPRO and against the protein families (Pfam) database [48]. tRNA genes were identified using tRNAscan-SE version 2.0 [49]. Genome assembly and annotation were performed sequentially on all phage genomes using the MEGAnnotator2 pipeline [50].

### Comparative genomic analysis of *Skunavirus* members

All bioinformatic analyses were performed using the MobaXterm server (https://mobaxterm.mobatek.net/). Genome sequences of *Skunavirus* members from Dutch dairy factories were retrieved from the NCBI database and included phages isolated in this study as well as those previously described (Table S3) [10,39, 42, 43, 51]. For protein-level comparisons, the markov clustering (MCL) algorithm was performed in the mclblastline pipeline v12-0678 with cut-off values of 50% AA identity across 50% coverage using an all-against-all bi-directional blast alignment, as described previously [13]. The produced MCL matrix was visualized and analysed using a two-way hierarchical clustering (HCL) in Orange v3.37 [52]. Different phage groups were identified based on MCL clusters.

### Classification of HNHE

The annotated 268 HNHEs from the assessed *Skunavirus* phages were subjected to HHPred (hidden markov model to hidden markov model prediction) analysis (https://toolkit.tuebingen.mpg.de/tools/hhpred), and the protein with the highest probability match to the subject was identified. The identified HNHEs were then classified as HmuI-like, PacI-like, GVE2-like or truncated.

To determine approximate insertion locations of each HNHE-encoding gene in a given *Skunavirus* genome, we first identified the genes encoding fully conserved or core proteins of lactococcal *Skunavirus* phages based on the orthologous clusters in the MCL analysis. The approximate insertion location of each HNHE-encoding gene was then derived based on the position of the nearest core protein-encoding genes (i.e. located either immediately upstream or downstream of each HNHE-encoding gene). Their nucleotide sequence identity to corresponding genes in the *Skunavirus* sk1 genome [53] was assessed by blastn search using the NCBI blast tool (https://blast.ncbi.nlm.nih.gov/).

AA sequences of all 121 I-HmuI-like HNHEs were aligned using MAFFT v.7 (https://mafft.cbrc.jp/alignment/server/index.html). Phylogenetic analysis was performed by the neighbour-joining method in Mega (v.12) [54], and the unrooted phylogenetic tree was visualized using ITOL v7 (https://itol.embl.de/).

### Protein sequence alignments

AA sequences of representative I-HmuI-like HNHEs from each identified insertion location (*H1* and *H3–H11*) were aligned using clustalw [55] in Mega (v11) and imaged with ALIGNMENTVIEWER (https://alignmentviewer.org/), i.e. protein identifiers (IDs; if applicable) or gene locus tags: ALM64117.1 (from phage 4.2), ALM63048.1 (from phage 4), A.1_22 (from phage A.1), ALM64149.1 (from phage 4.2), ALM64155.1 (from phage 4.2), ALM64160.1 (from phage 4.2), ALM64165.1 (from phage 4.2), ALM64616.1 (from phage E1127), M19_49 (from phage M19) and 7.1 n_24 (from phage 7.1 n).

### Computational structure prediction and analysis of HNHEs

Protein structures of selected I-HmuI-like HNHEs were predicted using AlphaFold3 [56] on a Google server (https://golgi.sandbox.google.com) by introducing the sequence of the HNHE protein, a Zn^2+^ ion, and a random dsDNA with a length between 26 and 32 bp. The associated images were created employing ChimeraX [57]. Coordinates of the predicted structures are accessible on Zenodo (https://zenodo.org/records/13767314).

### *In vivo* genetic exclusion activity assay

To test the exclusion activity of HNHEs against *Skunavirus* genomic DNA, seven HNHE-encoding genes (six I-HmuI-like and one PacI-like HNHEs) associated with different insertion locations or HNHE families were cloned into the nisin-inducible expression plasmid pPTPi [58], i.e. genes with locus tags: *Phi42_02* (from phage 4.2), *Phi93_03* (from phage 93), *Phi4_09* (from phage 4), *M51_28* and *M51_51* (from phage M51), *7 .p3_37* (from phage 7 .p3) and *PhiE1127_59* (from phage E1127). DNA of each phage was prepared, and HNHE-encoding genes were amplified by PCR using gene-specific primers (Table S2), containing restriction enzyme recognition sequences (SalI or BglII). PCR amplifications were performed using a 2720 Thermal Cycler (Applied Biosystems, Waltham, MA, USA) employing the following conditions: 5 min at 94 °C followed by 40 cycles (30 s at 94 °C, 30 s at 55 °C, 30 s at 72 °C) followed by a final extension step at 72 °C for 7 min. Following restriction enzyme treatment, the individual PCR products were ligated with plasmid pPTPi, which had been treated with the corresponding restriction enzymes, using T4 DNA ligase (Fisher Scientific, USA), according to the manufacturer’s instructions. The ligation mixture was introduced into competent *L. cremoris* NZ9000 by electroporation (20 µF, 200 Ω, 2.5 KV) followed by recovery for 2.5 h at 30 °C in GM17 (M17 supplemented with 0.5% glucose) with 20 mM MgCl_2_ and 2 mM CaCl_2_. Transformants were selected on GM17 agar plates supplemented with 10 µg ml^−1^ tetracycline (final concentration) and incubated overnight at 30 °C. The integrity of the generated constructs was confirmed by Sanger Sequencing by Azenta/GENEWIZ (Leipzig, Germany).

For culture preparations of host bacteria harbouring the nisin-inducible plasmid pPTPi or its derivatives, a fresh overnight culture with 10 µg ml^−1^ tetracycline was used to inoculate (1%) fresh growth medium (without tetracycline) and incubated until the optical density at 620 nm reached 0.2, followed by nisin (Sigma, USA) induction (through the addition of nisin at a final concentration of 20 ng ml^−1^) for 3 h at 30 °C. A 300 µl aliquot of the prepared bacterial culture was inoculated into 5 ml of GM17 semi-agar supplemented with CaCl_2_ (10 mM final concentration) and 100 µl of serial dilutions (10^0^–10^−7^) of phage sk1 (*Skunavirus*) [53]. The mixture was then plated onto GM17 agar, which was similarly supplemented with CaCl_2_, to perform a standard plaque assay procedure using the double-agar overlay approach [46]. Plaque formation was observed, following overnight incubation at 30 °C. The efficiency of plaquing (EOP) was calculated using NZ9000/pPTPi as a positive control. To assess the specificity of HNHE against *Skunavirus*, phage c2 (*Ceduovirus*) was included as a comparator [59]. Three biological repeats of each assay were performed.

## Results

### Bacteriophage isolation and identification

Previously, a large collection of *Skunavirus* members had been isolated from samples associated with Dutch dairy facilities. In order to expand this phage collection, a total of 103 dairy fermentation-derived samples were screened for bacteriophages (Table S1) using strains isolated from the corresponding starter culture, for example, 34 samples obtained when the cheese factory used starter culture SM1 were screened against lactococcal host strains named A through to T [42]. For samples without information regarding the corresponding starter culture, a range of *L. cremoris/L. lactis* strains representing different cell wall polysaccharide (CWPS) types (from SM1, SM2 and SM3 – A, B, C_1_, C_2_, C_4_ and unknown types) and reference strains (NZ9000 and 3107) were used for phage identification (Table S4). The CWPS of *Lactococcus* strains is known to serve as the primary receptor for specific binding of *Skunavirus* phage receptor-binding proteins (RBPs), thus determining the host range of a given phage [39,60]. To avoid isolating identical phages, a single representative phage infecting a distinct host strain was selected from individual samples of each production date or sample type (Table S1). Among the SM1-associated samples, 15 out of 34 tested positive for phage infection against at least one strain, and 14 phages were successfully isolated and propagated to high titres (~10^7^ PFU ml^−1^). For SM2, 18 out of the 19 assessed samples were phage-positive, resulting in the isolation of eight phages. For samples where information regarding the employed starter culture was unavailable, it was shown that all tested positive for phage infection, yielding 34 phage isolates. Multiplex PCR genotyping analysis of the isolated lactococcal phages revealed that all identified phages are members of the genus *Skunavirus* (data not shown). Based on host range analysis and other sample information, presumed identical phage isolates from each starter culture sample were excluded. In this manner, a total of 18 apparently distinct phage isolates were selected from screened dairy samples associated with fermentations employing starter cultures SM1 (*n*=9), SM2 (*n*=2) or an unknown starter culture (*n*=7) (Table 1).

**Table 1.** Bacteriophage isolated from dairy samples in this study

Starter originally used to obtain sample	Bacteriophage	Host strain used for isolation	CWPS type of host
SM1	A.1	A	C_1_
	L.1	L	Unknown
	D.1	D	C_1_
	B.1	B	A
	A.2	A	C_1_
	D.2	D	C_1_
	I.2	I	C*
	B.2	B	A
	N.2	N	C*
SM2	7.1	7	C_1_
	19.2 n	19	C_1_
Unknown	2 .p9	2	B
	7 .p3	7	C_1_
	L.l25	L	Unknown
	R.l24	R	C*
	L1.p3	L1	A
	Mm14.p9	Mm14	C_2_
	SB31.p3	SB31	C_4_

*Indicates the strains for which the C subtype was not determined.

### Diversity of isolated *Skunavirus* members and correlation to starter, geographical location, or other sample/isolation characteristics

The genomes of the 18 newly isolated phages were sequenced, revealing that, as expected, all represent genetically distinct phages (Table S5). These 18 newly isolated and sequenced phages were categorized together with 71 previously sequenced *Skunavirus* phages (derived from Dutch cheese whey), retrieved from the NCBI database (Table S3), based on their corresponding starter cultures (SM1, SM2, SM3 and unknown), associations with specific factories (F1, F2, F3, F4 and unknown) and year of isolation (2009, 2013, 2015, 2021 and 2024), if applicable. Genome sizes ranged from 27,529 to 34,004 bp, encompassing 48–63 ORFs and possessing a G+C content ranging between 34.43 and 35.52 mol% (Table 2). Notably, phages isolated from F3, which used starter culture SM3, encoded an average of 3 or 4 additional ORFs and demonstrated a larger genome size (of ~1,000 bp), with the lowest G+C content (34.81 mol%), compared with phages associated with other starter cultures or factories.

**Table 2.** Genetic features of *Skunavirus* phages isolated from dairy factory in the Netherlands

Factor		No. of phage	No. of ORF			Genome size (bp)			G+C (mol%)		
			Avg.	Max.	Min.	Avg.	Max.	Min.	Avg.	Max.	Min.
**Starter**	**SM1**	29	54	59	49	30,262	34,004	28,349	35.03	35.31	34.58
	**SM2**	41	57	63	52	31,302	33,017	28,811	34.81	35.21	34.43
	**SM3**	3	53	55	49	30,039	31,359	28,027	35.18	35.52	34.86
	**Unknown**	16	54	60	48	30,250	32,900	27,529	35.04	35.46	34.62
**Factory**	**F1**	16	54	58	49	30,154	32,855	28,349	34.96	35.31	34.58
	**F2**	4	53	55	49	30,509	31,841	28,983	35.02	35.14	34.92
	**F3**	41	57	63	52	31,302	33,017	28,811	34.81	35.21	34.43
	**F4**	12	53	55	49	30,269	34,004	28,027	35.17	35.52	34.86
	**Unknown**	16	54	60	48	30,250	32,900	27,529	35.04	35.46	34.62
**Year**	**2009**	17	54	58	49	30,686	32,150	28,437	34.88	35.25	34.58
	**2013**	22	55	63	49	30,371	32,682	28,349	34.91	35.46	34.49
	**2015**	29	57	61	51	31,432	33,017	28,732	34.87	35.3	34.43
	**2021**	19	53	57	48	30,192	34,004	27,529	35.13	35.52	34.82
	**2024**	2	53	54	52	30,041	30,065	30,017	34.84	34.90	34.78

Avg, average; Max, maximum; Min, minimum.

**Fig. 2.**
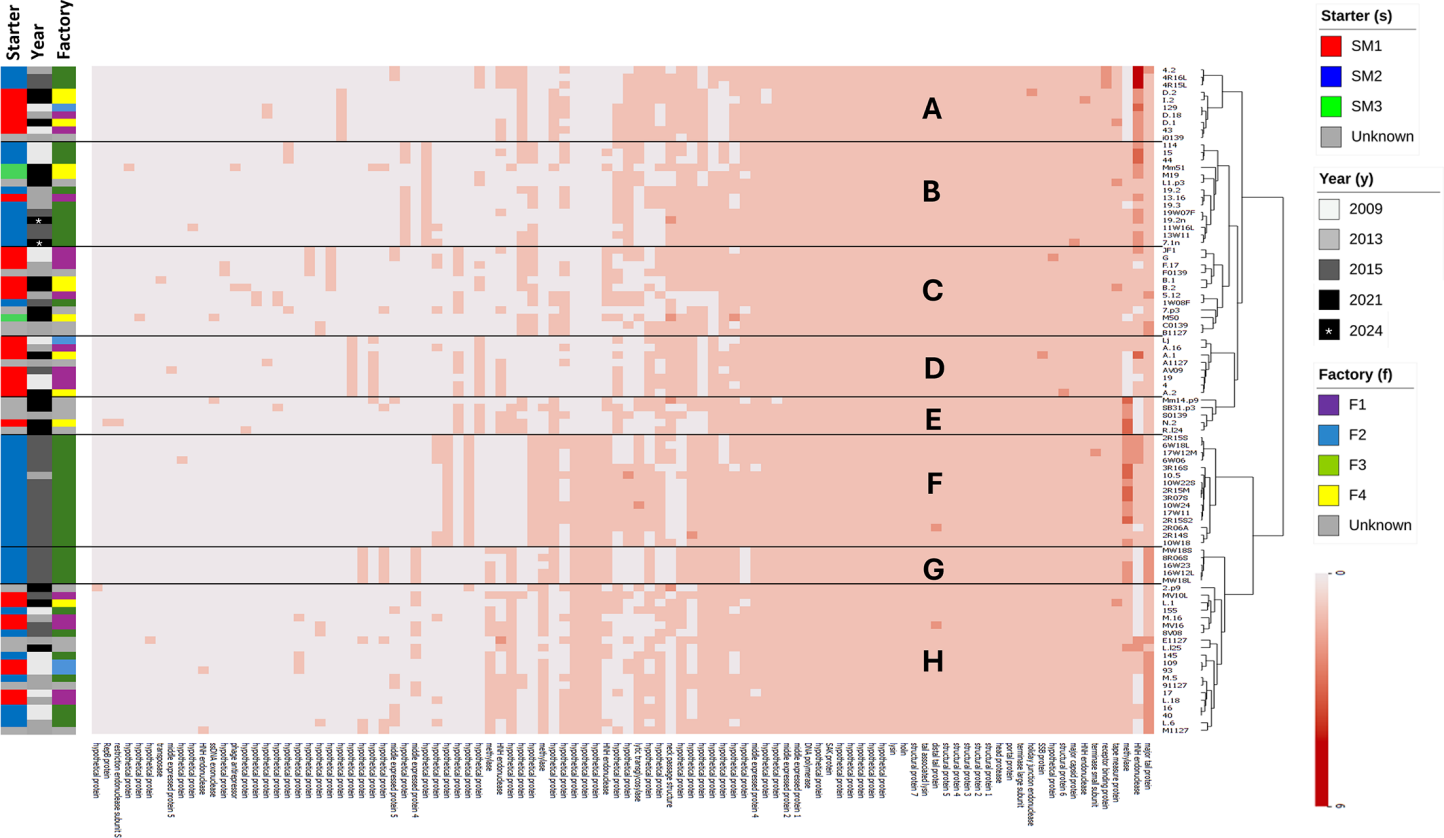
HCL of gene content variation among 89 *Skunavirus* phages isolated from Dutch dairy factories. The colour intensity of the bar at the bottom right indicates the number of genes encoding the corresponding proteins.

HCL of phage-derived proteomes was performed using the genome sequences of the 89 assessed Dutch *Skunavirus* members, based on the absence or presence of orthologous protein clusters (applied criteria: at least 50% AA identity with at least 50% protein coverage) (Fig. 2). This analysis identified eight distinct phage clusters (designated here as clusters A through to H). No clear cluster correlations were observed between phage clusters and the collection factors (starter cultures, factories and years). Nevertheless, phages isolated from samples using the same starter culture tended to cluster closely together, while phages isolated from the same factory or sampling year were scattered across different clusters. For example, cluster D is composed of starter culture SM1-associated phages, but these were isolated from various factories and across different years. Similarly, phage clusters A and C, which also contain SM1-derived phages, exhibit relatedness between gene content and the associated starter culture rather than factory origin or sample collection year.

### Classification of HNHE in *Skunavirus*

**Fig. 3.**
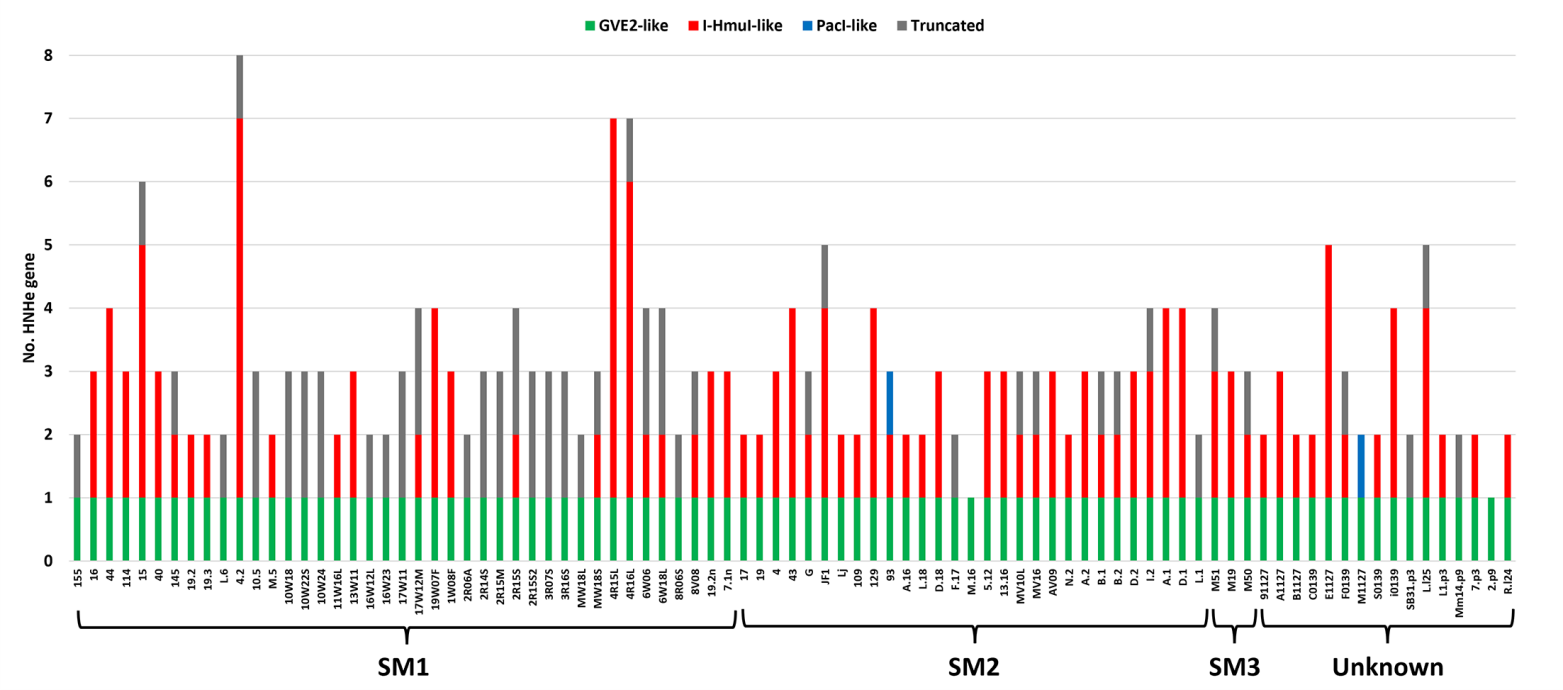
Identified HNHE-encoding genes from 89 *Skunavirus* genomes.

HNHE-encoding genes are frequently predicted to be present within *Skunavirus* genomes, and most have been presumed to represent homing endonucleases, potentially facilitating phage genetic exchanges [21,26]. To understand their prevalence and relationship to phage genomic diversity, HNHE-encoding genes were identified by blast-based alignments (Fig. 2). This analysis identified a total of 268 predicted HNHE-encoding genes spread across 89 assessed phage genomes. Based on the lack of intervening sequences between a given HNHE-encoding gene and its adjacent gene, it appeared that none of the HNHE-encoding genes is encoded within a self-splicing intron (as shown in Fig. 1) [20,61]. The number of HNHEs encoded by a given *Skunavirus* genome ranged from 1 to 8 (Fig. 3). Further AA sequence-based analysis using HHPred indicated that 212 of these HNHEs had significant similarity with one of three reference HNHEs, i.e. PacI [32], GVE2 [35] or I-HmuI [23]. In the current study, these identified *Skunavirus*-derived HNHEs were therefore classified into three distinct groups, i.e. I-HmuI-, PacI- or GVE2-like groups. Each examined *Skunavirus* phage was shown to encode a single GVE2-like HNHE (thus representing 89 HNHEs of the total 268 HNHEs identified), all positioned in the same relative genomic location. In contrast, the presence and number of I-HmuI- and PacI-like HNHE-encoding genes varied among the assessed phages. Especially, I-HmuI-like HNHE-encoding genes were most abundant, accounting for ~45% of all identified HNHE-encoding genes (121/268), whereas PacI-like HNHEs were seemingly rare, appearing only twice among all 89 assessed genomes (2/268). The remaining 56 genes, identified as HNHEs by BLASTP, appeared to represent incomplete HNHE proteins, suggesting they are partially deleted remnants of formerly complete HNHE-encoding genes. These presumably truncated HNHE-encoding genes were also shown to be variable in both presence and number across the assessed phage genomes (56/268).

To identify the approximate insertion location of HNHE-encoding genes within *Skunavirus* genomes, core proteins were determined based on the orthologous clusters (Fig. 2). Among them, their encoding genes located adjacent to each HNHE-encoding gene were identified. Most of these genes showed high nucleotide sequence similarities (>75%) to the corresponding genes of sk1, with at least 59% coverage (Table S6). The genes specifying the suppressor of AbiK (SAK)-like ssDNA annealing protein (encoded by *sk1p37*), hypothetical protein (encoded by *sk1p27*) and DNA polymerase (encoded by *sk1p45*) showed high sequence similarities (>75%), yet with limited coverages (26%, 37% and 15%, respectively).

**Fig. 4.**
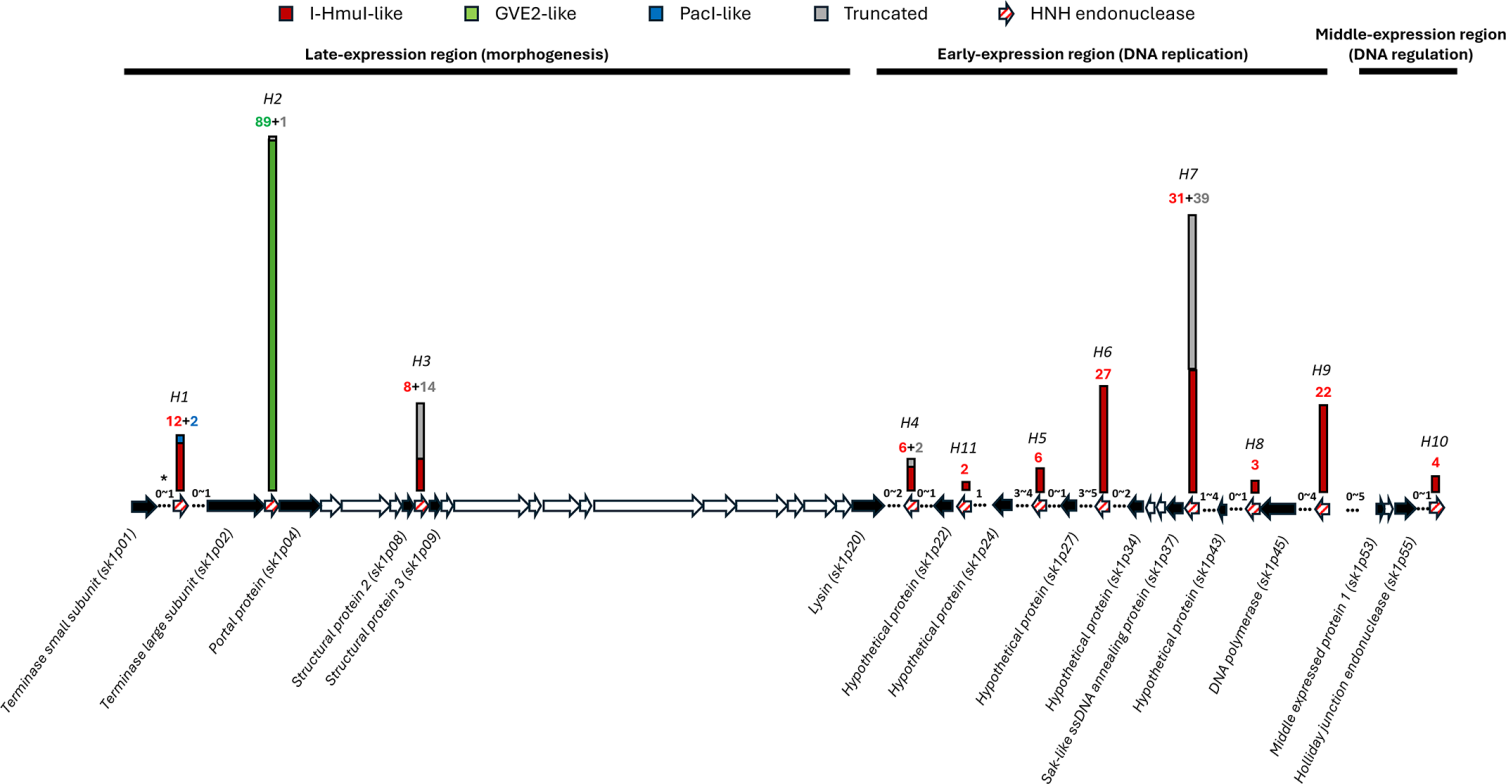
Schematic overview showing approximate insertion locations (H1–H11) of HNHE-encoding genes across *Skunavirus* genomes. *Denotes the number of genes between the HNHE-encoding gene and its core protein-encoding genes (black coloured). The number above the bar indicates the count of HNHE-encoding genes in each homology group (red: I-HmuI-like; green: GVE2-like; blue: PacI-like) or the number of truncated genes (grey).

Based on these core protein-encoding genes, the approximate insertion locations of HNHE-encoding genes within *Skunavirus* genomes were mapped (Fig. 4) to a total of 11 genomic positions (termed *H1* through to *H11*) across the 89 genomes. The I-HmuI-like HNHE-encoding genes were dispersed across ten of these identified insertion locations (the exception being *H2*) in these *Skunavirus* genomes, spanning the three functional modules of their genomes (these are related to the late-, early- and middle-expressed transcriptional units associated with morphogenesis, replication and regulation, respectively). Conversely, PacI- and GVE2-like HNHE-encoding genes were each found at a specific insertion location (*H1* and *H2*, respectively) in the morphogenesis module. All 89 GVE2-like HNHEs were encoded between the terminase large subunit (TerL)- and portal protein-encoding genes, which are involved in phage DNA packaging, indicating that this HNHE (inserted at *H2*) plays a role in DNA packaging that may be unrelated to homing [26], and we named this HNHE ‘HNHE related to DNA Packaging’ (HrdP). Most of the I-HmuI-like HNHE-encoding genes (97 out of 121) were identified in the replication module, distributed across seven locations (*H4–H9* and *H11*) (Fig. 4). Notably, insertion locations *H6*, *H7* and *H9* harboured 70 out of the 97 I-HmuI-like HNHE-encoding genes. Furthermore, most of the truncated HNHE-encoding genes were found at location *H7* (39/56).

### Relationship between HNHE and phage diversity

**Fig. 5.**
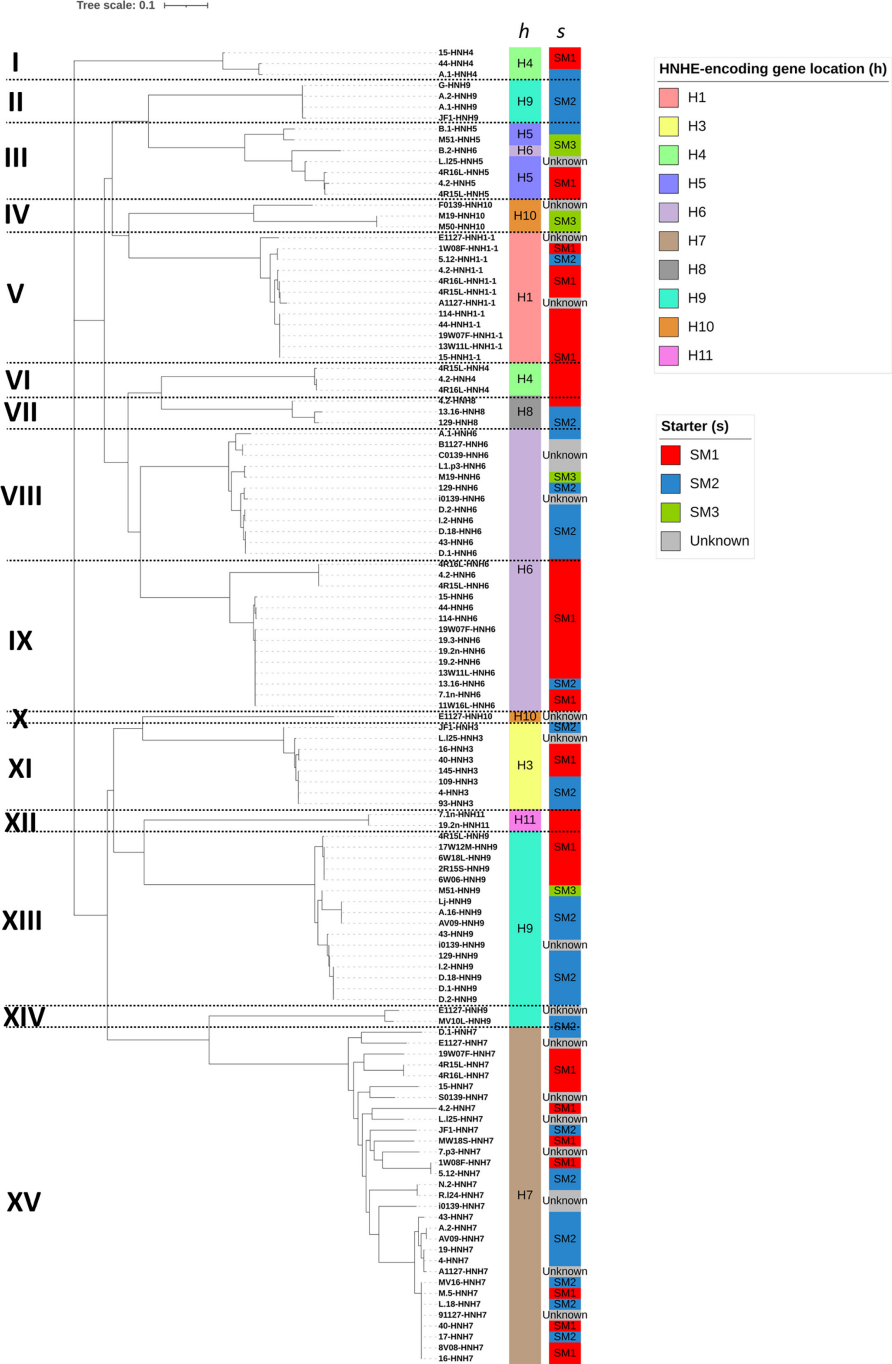
Phylogenetic comparison of I-HmuI-like HNHEs identified from Dutch *Skunavirus* genomes, with bootstrap values from 1,000 replicates.

To elucidate the relationship between phage diversity and homing endonucleases, a phylogenetic comparison of *Skunavirus*-associated I-HmuI-like HNHEs, which represent the most abundant and variable HNHEs among the three HNHE groups (i.e. I-HmuI-, PacI- and GVE2-like) identified in this study, was performed (Fig. 5). In total, 15 (designated here as clusters I through to XV) distinct phylogenetic clusters were identified. Interestingly, the insertion locations of HNHE-encoding genes were associated with these clusters, with each cluster representing a single insertion location, except for cluster III. Notably, insertion locations *H4*, *H6*, *H9* and *H10* were observed in multiple clusters, suggesting that distinct HNHEs can be inserted at the same insertion location within a *Skunavirus* genome.

Furthermore, members of a given phylogenetic cluster were shown to be frequently associated with the starter culture used for production. For example, clusters II, IV, V, VI, VIII, IX and XII were predominantly derived from phage isolates from a single, specific starter culture, indicative of a correlation between a given HNHE and its associated host strain. Conversely, HNHEs encoded by phages belonging to cluster XV are not specifically associated with a particular starter culture, as they were found in *Skunavirus* genomes derived from both starter SM1 and SM2 samples [17].

### Sequence alignment and structure prediction of HNHEs

**Fig. 6.**
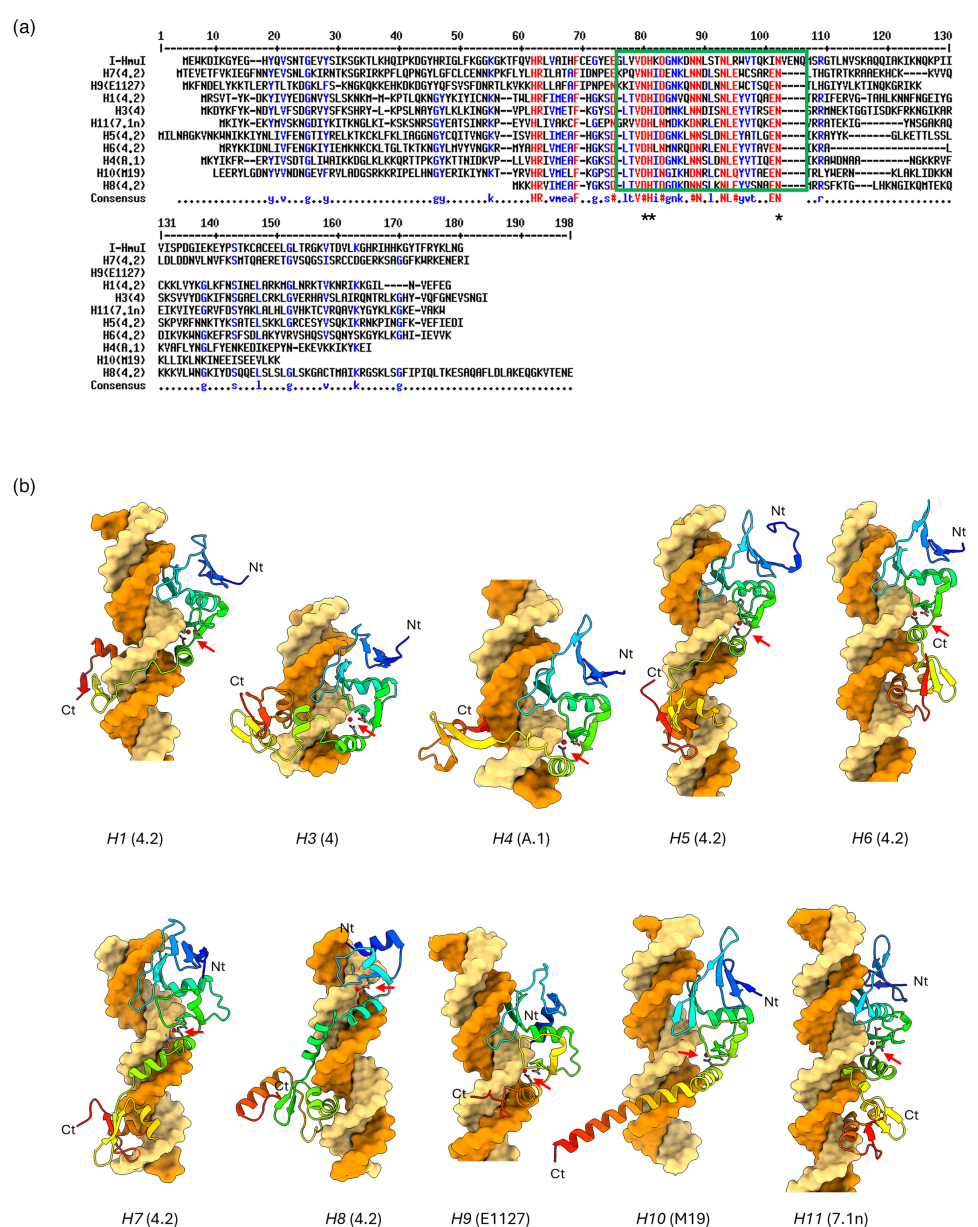
The structure of I-HmuI-like HNHEs from *Skunavirus*. (**a**) AA sequence alignment of I-HmuI-like HNHEs at each insertion location (*H1*, *H3*, *H4*, *H5*, *H6*, *H7*, *H8*, *H9*, *H10* and *H11*) with I-HmuI. The HN(H/N) catalytic domains are boxed in green, and catalysis associated residues are marked with asterisks. (**b**) Predicted protein structures of I-HmuI-like HNHEs. The Zn^2+^-binding residues are displayed as sticks, and the Zn^2+^ ion is represented as a red sphere and is indicated by red arrows. The dsDNA is coloured yellow and orange. The origin phage of each HNHE is indicated in parentheses. Nt, N-terminal; Ct, C-terminal

To explore the diversity of HNHE-encoding genes based on their insertion locations, the deduced protein sequences and predicted structures of representative I-HmuI-like HNHE-encoding genes from each insertion location (*H1*-4.2, *H3*-4, *H4*-A.1, *H5*-4.2, *H6*-4.2, *H7*-4.2, *H8*-4.2, *H9*-E1127, *H10*-M19 and *H11*-7.1n) were analysed. For this comparative analysis, PacI- and GVE2-like HNHEs were excluded, as all members of these families are encoded at the same insertion locations (*H1* and *H2*, respectively) (Fig. 4). Sequence alignment revealed that the catalytic domains, known as the HN(H/N) motif, exhibited high similarity with conserved catalytic residues (D74, H75 and N96 of I-HmuI) (Fig. 6a) [23]. The C-terminal region of HNHEs was relatively variable, compared with the N-terminal region, including the active site.

Protein structure predictions using AlphaFold3 showed that all I-HmuI-like HNHEs are represented by two β-sheets and a single α-helix, with a Zn^2+^ ion-binding site in the N-terminal region, referred to as the ββα-metal active site, which is typically found in HNHEs (Fig. 6b) [62]. The predicted models reported interactions between two residues of each HNHE and the catalytic Zn^2+^ ion, as well as with dsDNA (Table S7). The N-terminal domain of all analysed HNHEs was shown to consist of a globular domain and to fit into the large groove of a dsDNA molecule, bringing the Zn^2+^ ion into the vicinity of the dsDNA backbone upper lip of the small groove. However, consistent with the sequence alignment, the C-terminal region of the HNHE protein structures exhibited variability, compared with the N-terminal region, indicative of (variable) DNA-binding specificity. Except for HNHE-encoding genes located at *H9* and *H10*, the C-terminal domains of the other HNHEs were also shown to encompass a globular domain.

### Marker exclusion activity of HNHE

**Fig. 7.**
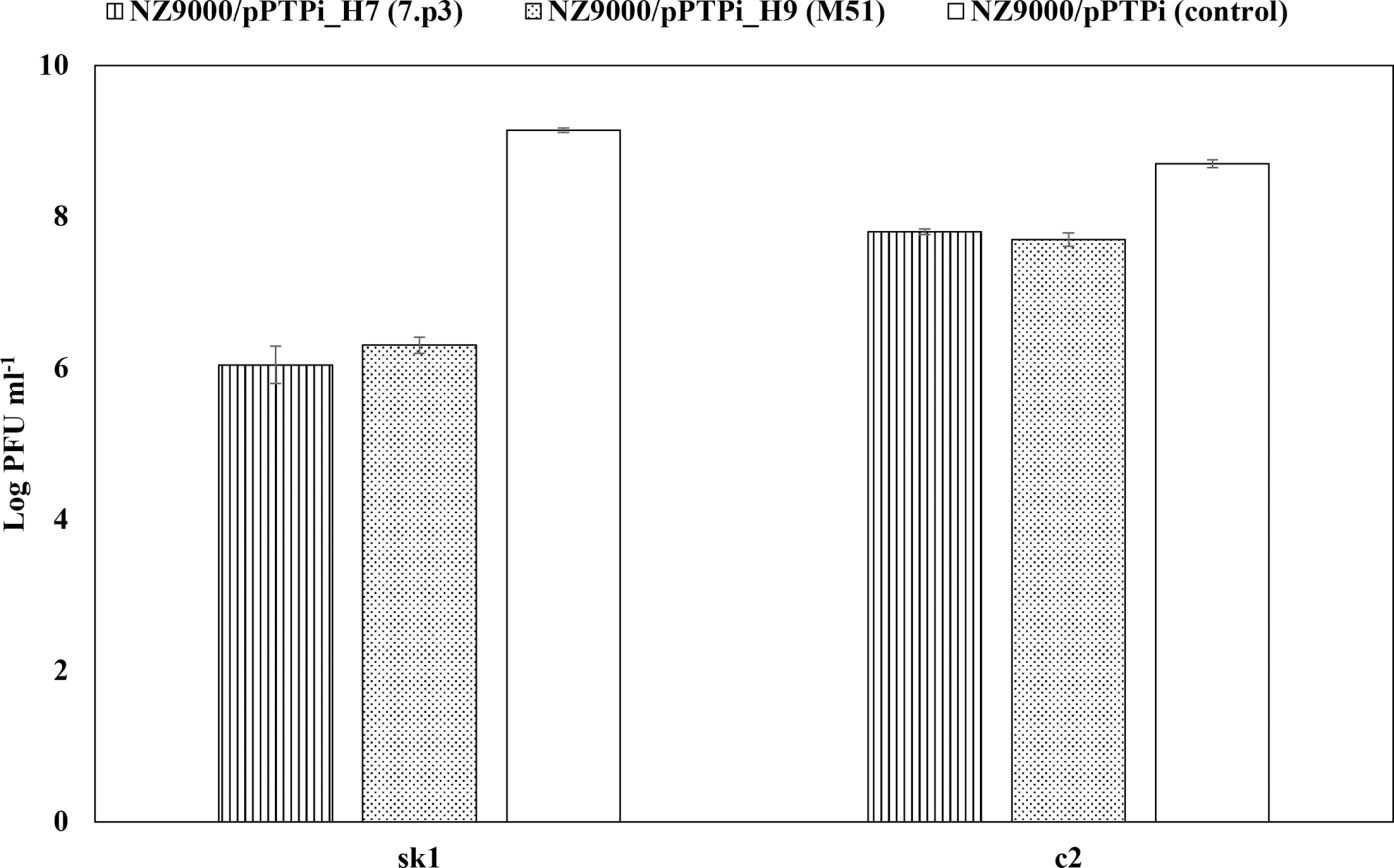
*In vivo* marker exclusion activity of HNHEs (*H7* from phage 7 .p3 and *H9* from phage M51) against *Skunavirus* and *Ceduovirus*.

Homing endonucleases are known to act as sequence-specific endonucleases to the homologous allele lacking their encoding genes [21]. The presumed specific endonucleolytic activities of HNHEs encoded by Dutch *Skunavirus* genomes were examined *in vivo* against *Skunavirus* sk1, which lacks these HNHE-encoding genes. Host bacteria (*L. cremoris* NZ9000) harbouring a particular HNHE-encoding gene (I-HmuI-like: *H1*-4.2, *H3*-4, *H5*-M51, *H7*-7.p3, *H9*-M51 or *H10*-E1127; PacI-like: *H1*-93) within a nisin-inducible plasmid (pPTPi) were induced with nisin and subsequently subjected to phage infection with *Skunavirus* sk1 or *Ceduovirus* c2, after which the (reduction of) EOP was calculated (Fig. 7) [53]. Compared with the control (NZ9000/pPTPi), which was shown to exhibit a titre of ~10^9^ PFU ml^–1^ of *Skunavirus* sk1, both HNH7 and HNH9 demonstrated ~10^3^-fold reduction in plaque numbers (EOP=0.0079 and 0.0014, respectively). The EOPs of *Ceduovirus* c2 on HNH7- and HNH9-expressing hosts were 0.12 and 0.10, respectively, suggesting that both HNHEs specifically target the *Skunavirus* genome. The other five HNHEs did not display significant activity against either sk1 or c2 (data not shown).

## Discussion

In this study, HNHEs encoded by 89 different genomes from *Skunavirus* members isolated from Dutch dairy fermentation facilities were identified and characterized. Among the 268 identified, apparently ‘free-standing’ HNHE-encoding genes (Fig. 3), 89 HNHEs presented homology with GVE2 HNHE [35]. These latter HNHEs, named HrdP, were encoded by all analysed *Skunavirus* genomes, and their corresponding gene was shown to be located between the terminase large subunit- and portal protein-encoding genes, indicating that they play a critical role in phage DNA packaging, as has been described for other phages [26,33, 62].

The remaining 179 HNHEs (i.e. I-HmuI-like, PacI-like and truncated) were shown to exhibit variable presence and frequency across different *Skunavirus* genomes, suggesting that these HNHEs play non-essential roles in phage fitness, likely functioning as homing endonucleases, as described previously [26]. Interestingly, the majority of these presumptive HNH homing endonucleases are encoded by genes present in the early-expressed region (126/179) (Fig. 4), an area known for its high genetic diversity in *Skunavirus* genomes [14,16]. Especially, these HNHE-encoding genes were the most frequently observed at insertion locations *H6*, *H7* and *H9*, and the number of flanking genes between their insertion locations and the nearest core protein-encoding genes was shown to be more variable compared with others. This suggests that homing events may occur frequently near these insertion locations. Additionally, their core protein-encoding genes showed lower sequence coverage/identity levels compared with corresponding genes from *Skunavirus* sk1, compared with other core proteins. These DNA polymorphisms may result from homing events, which influence the sensitivity of DNA against the corresponding homing endonuclease.

The phylogenetic comparison of I-HmuI-like HNHEs demonstrates the significant association between HNHE phylogeny position and their corresponding insertion location. As shown in Fig. 6, the HNHEs associated with each insertion location exhibit sequence and structural diversity in their C-terminal region, which is presumed to function as the DNA-binding domain. While sharing a conserved N-terminal HNH-motif, the structural diversity at the C-terminal region likely contributes to their functional differences in recognizing and cleaving specific phage genome sequences, which in turn is indirectly responsible for distinct insertion locations [23]. Furthermore, the apparent marker exclusion activities of particular HNHEs in nisin-induced host bacteria against *Skunavirus* infection are a clear indication of their sequence-specific endonucleolytic activities. These results suggest that HNHEs at each insertion location possess distinct target sequences and may drive genomic recombination within only specific parts of the genome among phages of the same genus.

Correlations between phage diversity and their host range have been elucidated in previous studies [17,63]. Our findings are consistent with these observations, as presented by certain MCL groups showing an association with the specific starter cultures used in the dairy samples. Similarly, most of the phylogenetic clusters of I-HmuI-like HNHEs show associations with starter cultures (Fig. 5). Since the inheritance of a HEG is known to occur during co-infection of host bacteria, these observations might be attributed to the strain-level host range of *Skunavirus*. Possibly, a particular host strain composition (e.g. containing strains with specific CWPS types) of a given starter culture may create different phage populations possessing particular HEGs. This may result in the inheritance or distribution of specific HEGs among phages associated with particular starter cultures, potentially influencing phage diversity. On the other hand, as shown by phylogenetic clustering, cluster XV, which is specific to the insertion location (*H7*), did not show a significant association with the starter culture. This lack of association may be attributed to the presence of host strains in both starter cultures (SM1 and SM2), which could be susceptible to homologous phages.

In conclusion, we identified and characterized HNHE-encoding genes in various *Skunavirus* genomes, which may be linked with *Skunavirus* genome diversity. Our work provides a valuable ‘roadmap’ for further investigation into the role HNHEs may play in *Skunavirus* genetic exchanges. For future research, a detailed investigation of individual HNHE insertion locations will be required to better understand this process. Through screening of mutant phages that have become insensitive to HNHE activity, escape mechanisms from this endonuclease activity, possibly through mutations in the target sequence, can be elucidated. This will provide a clearer understanding of the specific target sequences for each HNHE. Furthermore, experimental studies are necessary to confirm whether these HNHEs actively promote homing events in *Skunavirus* phages, thereby enhancing their genetic diversity. These insights will improve our understanding of their role in genome evolution.

## Supplementary material

10.1099/mgen.0.001548 Uncited Fig. S1.

## Abbreviations

AA: amino acid
CWPS: cell wall polysaccharide
EOP: efficiency of plaquing
HCL: hierarchical clustering
HEG: homing endonuclease-encoding gene
HNHE: HNH endonuclease
HrdP: HNHE related to DNA Packaging
MCL: Markov clustering
NCBI: National Center for Biotechnology Information
RBP: receptor-binnding protein
