# Supplementary material for: Diversity and functional characterization of HNH endonucleases encoded by lactococcal Skunavirus phages
Source: Microb Genom. 2025 Dec 23;11(12):001548. doi: 10.1099/mgen.0.001548 (PMC12724721; doi:10.1099/mgen.0.001548)
Supplement: Uncited Fig. S1. [file mgen-11-01548-s001.pdf]

Supplementary materials

Table S1. Dairy samples analysed in this study.

Starter	Factory	Collection time		Sample type (number)
		Year	Date/Month	
SM1	F5	2021	4.August	Ingredients (i.e. milk, starter culture, and whey cream) (5), whey (9), and curd (3)
			23.September	Ingredients (i.e. milk, starter culture, and whey cream) (5), whey (9), and curd (3)
SM2	F3	2024	20.April	Whey (10)
			21.April	Whey (9)
Unknown	Unknown	2021	-	Whey powder (16) and whey concentrate (34)

Table S2. Primers used in this study.

Target locus[a]		Primer	Sequence (5' to 3')[b]	Amplicon size (bp)	Reference
**Lactococcal phage genotyping**					
Lactococcal phage group	*Skunavirus*	936A	TCAATGGAAGACCAAGCGGA	179	Labrie et al., 2000
		936B	GTAGGAGACCAACCCAAGCC		
	Ceduovirus	c2A	CAGGTGTAAAAGTTCGAGAACT	474	
		c2B	CAGATAATGCACCTGAATCA		
	P335-like phage	P335A	GAAGCTAGGCGAATCAGTAA	682	
		P335B	GATTGCCATTTGCGCTCTGA		
**HNHE-encoding gene cloning**					
H1 (4.2)		SalI_H1_4.2_F2	AGCAGCGTCGAC**AGGAGGCACTCACC**ATGAGAAGTGTAACTTATAAAGAT	500	This study
		BglII_H1_4.2_R	AGCAGCAGATCTTTATCCCTCGAATTCTACATTC		
H1 (93)		SalI_H1_93_F	AGCAGCGTCGAC**AGGAGGCACTCACC**ATGATTGATTTAACTAATAAGAAATACG	602	
		BglII_H1_93_R	AGCAGCAGATCTTTATTTACAATAATGTTCATAAACTCTC		
H3 (4)		SalI _H3_4_F	AGCAGCGTCGAC**AGGAGGCACTCACC**ATGAAAGATTATAAATTTTATAAAAATGATTATTTAG	536	
		BglII _H3_4_R	AGCAGCAGATCTTCAAATTCCATTACTTACCTCG		
H5 (M51)		SalI _H5_M51_F	AGCAGCGTCGAC**AGGAGGCACTCACC**ATGGAATATAAAAAAATATATAATTTAATTGTTTTC	506	
		BglII _H5_M51_R	AGCAGCAGATCTTTATATAAATTCAACTTTAAAACCATTAATC		
H7 (7.p3)		SalI _H7_7.p3_F	AGCAGCGTCGAC**AGGAGGCACTCACC**ATGAACGAAGTTGAAACTTTTGTTAA	539	

	BglII _H7_7.p3_R	AGCAGCAGATCTTCATTTTTTCATCCATTTATAGCC	
H9 (M51)	SalI _H9_M51_F	AGCAGCGTCGAC**AGGAGGCACTCACC**ATGAAAGTTGAACAAAAAATATATAAAGG	530
	BglII _H9_M51_R	AGCAGCAGATCTTTATATTTTTTCTATTTTTGTTCCTTTTAATTTAC	
H10 (E1127)	SalI _H10_E1127_F	AGCAGCGTCGAC**AGGAGGCACTCACC**ATGCTAAGCAAAATTTATAAAAATGATTATA	506
	BglII _H10_E1127_R	AGCAGCAGATCTTTATTTATCCTGACTTTCTACATAG	

[a]The origin phage of gene is indicated within the brackets. [b]Restriction enzyme sites for ligation are underlined, and ribosomal RNA binding sites are highlighted in bold.

Table S3. Lactococcal bacteriophages belonging to *Skunavirus* isolated from Dutch dairy factories.

Starter	Factory	Year	Sample type (number)	Phage	Accession number	Reference
SM1	F1	2009	Whey (10)	4	KP793101	Murphy et al., 2013
				19	KP793103	
				17	KP793114	
				43	KP793110	
				G	KP793117	
				JF1	KP793129	
		2013	Whey (12)	A.16	KP793102	Murphy et al., 2016
				L.18	KP793120	
				D.18	KP793107	
				5.12	KP793108	
				13.16	KP793116	
				M.16	KP793128	
				F.17	KP793113	
		2015	Whey (18)	AV09	MK301439	N/A
				MV10L	MK301440	
				MV16	MK301441	
	F2	2009	Whey (12)	109	KP793121	Murphy et al., 2013
				93	KM091443	
				129	KP793112	
				Lj	KP793133	
	F5	2021	Ingredients (i.e. milk, starter culture, and whey cream) (10), whey (18), and curd (6)	A.1	PQ675582	This study
				L.1	PQ675589	
				D.1	PQ675586	
				B.1	PQ675584	
				A.2	PQ675583	

				D.2	PQ675587	
				I.2	PQ675588	
				B.2	PQ675585	
				N.2	PQ675590	
SM2	F3	2009	Whey (12)	155	KP793130	Murphy et al., 2013
				16	KP793135	
				44	KP793124	
				114	KP793115	
				15	KM091442	
				40	KP793127	
				145	KM091444	
		2013	Whey (12)	19.3	KP793105	Murphy et al., 2016
				L.6	KP793122	
				4.2	KP793123	
				10.5	KP793119	
				M.5	KP793126	
				19.2	KP793111	
		2015	Whey (40)	1W08F	MK301437	N/A
				8V08	MK301438	
				10W18	KX379664	Hayes et al., 2018
				10W22S	KX379669	
				10W24	KX346245	
				11W16L	KX346246	
				13W11L	KX379671	
				19W07F	KX379670	
				6W06	KX346243	
				6W18L	KX346244	
				16W12L	KX379672	

				16W23	KX346249	
				17W11	KX346247	
				17W12M	KX346248	
				MW18L	KX379673	
				MW18S	KX346250	
				2R14S	KX346236	
				2R15M	KX346237	
				2R15S	KX346238	
				2R15S2	KX346239	
				2R06A	KX346240	
				3R07S	KX346241	
				3R16S	KX379666	
				4R15L	KX379667	
				4R16L	KX379668	
				8R06S	KX346242	
		2024	Whey (19)	7.1n	PQ675592	This study
				19.2n	PQ675591	
SM3	F5	2021	Ingredients (i.e. milk, starter culture, and whey cream) (10), whey (18), and curd (6)	M19	PQ675600	Yu et al., 2025
				M50	PQ675601	
				M51	PQ675602	
Unknown	F4	2013	Whey cream (9)	F0139	KP793118	Murphy et al., 2016
				A1127	KP793106	
				C0139	KP793109	
				91127	KP793125	
				B1127	KP793104	
				S0139	KP793134	
				E1127	KP793131	
				M1127	KP793132	

			i0139	KX379665	Hayes et al., 2017
Unknown	2021	Whey powder (16) and concentrate (34)	2.p9	PQ675593	This study
			7.p3	PQ675594	
			L.l25	PQ675595	
			L1.p3	PQ675596	
			Mm14.p9	PQ675597	
			R.l24	PQ675598	
			SB31.p3	PQ675599	

*N/A: Not applicable.

Table S4. *Lactococcus cremoris/lactis* strains used for bacteriophage screening from unknown starter samples.

Host strains	CWPS type	Reference
A	C_1	Murphy et al., 2013
B	A	
C	A	
D	C_1	
F	C_1	
L	U	
N	C	
R	C	
1	C	
2	B	
4	C_4	
7	C_1	
16	U	
L1	A	Yu et al., 2025
L7	C_2	
Mm3	C_1	
Mm14	C_2	
R15	C_2	
SB31	C_4	
NZ9000	C_1	Linares et al., 2010
3107	C_2	Garzon et al., 2019

Table S5. Genetic information of *Skunavirus* phages isolated in this study.

Starter of samples	Bacteriophage	No. ORF	No. tRNA	GC (%)	Genome size (bp)
SM1	A.1	54	0	35.15	29123
	L.1	53	0	35.23	31184
	D.1	53	1	35.19	29683
	B.1	52	3	35.03	31152
	A.2	52	0	35.25	28682
	D.2	51	1	35.29	29018
	I.2	51	1	35.30	29018
	B.2	55	2	35.00	31242
	N.2	53	0	35.05	34004
SM2	7.1n	54	0	34.78	30065
	19.2n	52	0	34.90	30017
Unknown	2.p9	55	0	34.95	30766
	7.p3	48	0	34.91	29419
	L.l25	55	0	34.96	30325
	R.l24	53	0	35.33	29972
	L1.p3	50	0	35.22	27529
	Mm14.p9	55	0	35.25	32900
	SB31.p3	57	1	34.82	29514

Table S6. Genetic similarities of conserved genes from *Skunavirus* genomes.

**Presumptive genes containing target sequence of HNHEs (sk1 locus tag)**	**Min. identity (%)**	**Min. query coverage (%)**
Terminase small subunit (sk1p01)	92	76
Terminase large subunit (sk1p02)	91	79
Portal protein (sk1p04)	90	98
Structure protein 2 (sk1p08)	88	97
Structure protein 3 (sk1p09)	94	73
Lysin (sk1p20)	81	64
Hypothetical protein (sk1p22)	89	94
Hypothetical protein (sk1p24)	76	66
Hypothetical protein (sk1p27)	83	37
Hypothetical protein (sk1p34)	87	59
SAK-like ssDNA annealing protein (sk1p37)	75	26
Hypothetical protein (sk1p43)	85	89
DNA polymerase (sk1p45)	88	15
Middled expressed protein 1 (sk1p53)	93	100
Holiday junction endonuclease (sk1p55)	85	87

Table S7. Amino acid residues associated with Zn^{2+} ion binding from I-HmuI-like HNHEs.

HNHE insertion region (origin phage)	Residue 1	Residue 2
H1 (4.2)	Asp 65	Asn 87
H3 (4)	Asp 66	Asn 88
H4 (A.1)	Asn 67	Asp 89
H5 (4.2)	Asp 76	Asn 98
H6 (4.2)	Asp 66	Asn 88
H7 (4.2)	Asn 77	Asn 99
H8 (4.2)	Asp 20	Asn 42
H9 (E1127)	Glu 90	His 100
H10 (M19)	Asp 67	Asn 89
H11 (7.1n)	Asp 65	Asn 87
